# Inter- and Intra-observer Reliability of MRI for Lumbar Lateral Disc Herniation

**DOI:** 10.4055/cios.2009.1.1.34

**Published:** 2009-02-06

**Authors:** Seong Wan Kim, Jin S. Yeom, Seong Kyu Park, Bong Soon Chang, Dong-Ho Lee, Jae Hyup Lee, Kun-Woo Park, Eun Seok Seo, Choon-Ki Lee

**Affiliations:** Department of Orthopaedic Surgery, Seoul National University College of Medicine, Seoul, Korea.; * Department of Orthopaedic Surgery, Ulsan University College of Medicine, Seoul, Korea.

**Keywords:** Lumbar lateral disc herniation, Inter-observer reliability, Intra-observer reliability, Magnetic resonance imaging

## Abstract

**Background:**

The authors analyzed inter- and intra-observer agreement with respect to interpretation of simple magnetic resonance T1- and T2-weighted axial and sagittal images for the diagnosis of lumbar lateral disc herniation, including foraminal and extraforaminal disc herniations.

**Methods:**

Forty-two patients in whom lumbar lateral disc herniation was suspected or confirmed by simple magnetic resonance imaging at one institute between May 2003 and December 2004 were included. The magnetic resonance images consisting of T1- and T2-weighted axial and sagittal images, and these were reviewed blindly and independently by three orthopaedic spine surgeons in a random manner. The images were interpreted as positive or negative for lateral disc herniation on 2 different occasions 3 months apart. Results were analyzed using Cohen's kappa statistic, and strengths of agreements were determined using the Landis and Koch criteria.

**Results:**

The kappa values for inter-observer agreement averaged 0.234 (0.282, 0.111, and 0.308 respectively) on the first occasion, and 0.166 (0.249, 0.111, and 0.137 respectively) on the second occasion, with an overall mean value of 0.200. Thus, the strength of agreement was only slight-to-fair according to the Landis and Koch criteria. Kappa values for intra-observer agreement averaged 0.479 (0.488, 0.491, and 0.459 respectively), indicating moderate agreement.

**Conclusions:**

The present study indicates that simple magnetic resonance imaging is not a reliable imaging modality for diagnosing lumbar lateral disc herniation. Another imaging study with improved diagnostic values should be developed to diagnose this pathologic finding.

Lumbar lateral disc herniation (LDH) is defined as a herniated disc in which the main portion is located lateral to the medial margin of the ipsilateral pedicle.^1^,2) LDH may be subdivided into foraminal and extraforaminal disc herniations. The main portion of the former is located between the medial and lateral margins of the ipsilateral pedicle, while in the latter, it is located lateral to the lateral margin of the pedicle.2) Although modern magnetic resonance imaging (MRI) is known to be a reliable modality for diagnosing central and paracentral disc herniations, its reliability for the diagnosis of lumbar LDH has not been established. Thus, the present study was undertaken to determine the diagnostic value of simple MRI for the diagnosis of lumbar LDH based on analyses of inter- and intra-observer reliability.

## METHODS

### Materials

This study received the approval of our Institutional Review Board. All patients suspected or confirmed to have LDH by simple MRI at one institution between May 2003 and December 2004 were considered for this study, and only those patients who fulfilled the following criteria were selected. First, those with spinal stenosis or spondylolisthesis at the same level as LDH were excluded. Second, only those who had at least four series of MRI, including T1- and T2-weighted axial and sagittal images, were included. Finally, only those with at least four axial images available at the LDH level to visualize foraminal levels, as well as disc levels, and with sagittal images that included all foraminal areas and the medial portion of the extraforaminal areas at the level and side of the LDH were included. All included patients underwent MRI at a single hospital on one MRI scanner (Gyroscan Intera 1.5T, Philips Medical Systems, Best, The Netherlands). According to the standard protocol used, sagittal images included all foraminal areas and the medial portions of the extraforaminal areas on both sides; four to six axial images were available at each level. In five patients in whom this protocol was not fully adhered to, the MRI was repeated to visualize the foraminal zone and the medial portion of the extraforaminal zone at the sides and at the levels of the suspected lesions.

Among the 42 patients finally enrolled in this study, there were 9 males and 33 females. Their age averaged 65 years (range, 49 to 81 years). Eight, 11, and 23 patients had suspected or confirmed LDH at L3-4, L4-5, and L5-S1, respectively. Nineteen LDHs were located mainly in the extraforaminal zone and 23 LDHs were located primarily in the foraminal zone. Twenty-one of the 42 cases were confirmed as LDH by definite clinical findings with or without other studies, e.g., MR-discography or computed tomography-discography and/or surgical confirmation. Five of the 42 cases were initially suspected, but were later confirmed not to be LDH. In the other 16 cases, although LDH was suspected by simple MRI, LDH was not confirmed because the clinical findings were unclear and no further work-up or surgery was conducted. These 16 cases included those who refused to have further imaging studies or treatment, such as root blocks or surgery.

The MR images of all the included patients were retrieved from the server of a picture archiving and communication system (PACS) in digital imaging and communication in medicine (DICOM) format, and stored in a personal computer.

### Review and Interpretation of the MR Images

The MRIs of the 42 patients, comprised of T1- and T2-weighted axial and sagittal images, were reviewed blindly and independently by 3 orthopaedic spine surgeons, who were also assistant professors at university hospitals other than the hospital that treated the patients. Furthermore, the reviewers did not participate in either the care of the included patients or their selection. All 3 orthopaedic spine surgeons had completed a 1-year spine fellowship, and had subsequently practiced as independent spine surgeons for > 2, 4, or 6 years. The MR images were reviewed in a random sequence, irrespective of a definitive diagnosis or lesion location. The sequence was determined by a research assistant, who was unaware of any patient information, based on patient numbers. All MR images were stored in digital format and were reviewed on two 17-inch liquid crystal digital (LCD) monitors using Impax software (AGFA, Ridgefield Park, NJ, USA). The reviewers were able to freely adjust image brightness and contrast and zoom, and were able to compare sagittal and axial images simultaneously. In some of the included patients, other images, such as coronal images, MRI-discograms, and/or enhanced MR images, were obtained in addition to the simple MR images. However, the reviewers were not allowed to access these images, and were unaware of any clinical, diagnostic, or treatment-related information. The reviewers were only given information on the affected or suspected side and level (e.g., left L4-5), and were asked to decide, based on the MR images, whether the patients were positive or negative for LDH for the given level and side. All three orthopaedic spine surgeons reviewed the images independently and were allowed to change their minds at any time during the interpretation session, which took each interpreter several hours.

In addition, all 3 reviewers repeated this interpretative exercise using the same MRIs 3 months later to provide intra-observer reliability data. During this second review, no information related to previous results was allowed, and this second image review was organized in the same manner by a blinded research assistant.

### Statistical Analysis

The three paired inter- and three intra-observer comparisons were analyzed using Cohen's kappa statistics (SPSS ver. 15.0). The strengths of the inter- and intra-observer agreements were determined using the Landis and Koch criteria (Table 1).3)

## RESULTS

**Fig. 1:**
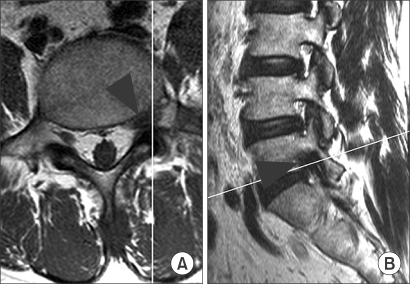
The axial (A) and sagittal (B) MR images of a 58 year-old man are shown. The white line in each figure indicates the sectioning plane for the other figure. A foraminal disc herniation on the left side at L5-S1 (arrowheads) was interpreted as positive for lateral disc herniation by all three observers during both image-reading sessions.

**Fig. 2:**
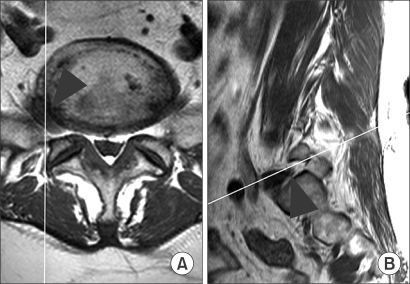
The axial (A) and sagittal (B) MR images of a 58 year-old woman are shown. The white line in each figure indicates the sectioning plane for the other figure. An extraforaminal herniation on the right side at L5-S1 (arrowhead) was suspected from the axial image (A). The sagittal image obtained in the extraforaminal zone (arrowhead) was not helpful due to the lack of reliable anatomical landmarks (B). These images led to different interpretations among the three observers during both reading sessions, i.e., positive, negative, negative during the first, and positive, negative, and positive during the second. This patient achieved symptom improvement by a L5 root block; no further imaging study was done.

**Fig. 3:**
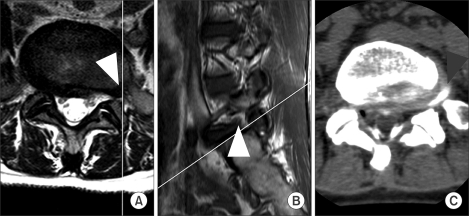
MR (A, B) and CT-discography (C) images of a 56 year-old woman with extremely severe radiating pain on the left side. The MR images (A, B) did not clearly show if the patient had an LDH at L5-S1 (arrow-heads). The interpretations of the three observers were negative, negative, and positive on the first reading session and negative, negative, and negative during the second. CT-discography (C) revealed an extrafor-aminal herniation at L5-S1 (arrowhead).

The reviewer interpretations were the same among the 3 reviewers for only 17 patients (40%) during the first review and for 14 patients (33%) during the second review (Fig. 1). Thus interpretations differed in > one-half of the cases (Figs. 2, 3). The kappa values for inter-observer agreement between the 3 interpreters were 0.282, 0.111, and 0.308 (average, 0.234) for the first review, and 0.249, 0.111, and 0.137 (average, 0.166) for the second review (Table 2). The overall average kappa value was 0.200, which indicated only slight-to-fair agreement, according to the Landis and Koch criteria.3)

The kappa values for intra-observer agreement were better than those for inter-observer reliability (0.488, 0.491, and 0.459 respectively; average, 0.479)(Table 3). The strength of agreement was moderate, according to the Landis and Koch criteria.3)

The kappa values for inter-observer agreement between the 3 interpreters averaged 0.282 at L3-4, 0.223 at L4-5, and 0.158 at L5-S1 (Table 4). The kappa values for inter-observer agreement averaged 0.521 at L3-4, 0.587 at L4-5, and 0.427 at L5-S1.

## DISCUSSION

MRI is the most reliable diagnostic method currently available for central and paracentral lumbar disc herniations, and accordingly, spine surgeons use MRI to diagnose these herniations and make therapeutic decisions. However, surprisingly little is known about the diagnostic value of MRI in LDH, and in order to determine whether simple MRI can adequately produce a definitive diagnosis, its reliability must be established.

In 1974, Abdullah et al.4) first demonstrated, by discography, an extreme lateral herniation of the lumbar disc compressing an exiting nerve root, which caused radiating pain. Subsequently, several authors described diagnostic imaging methods for LDH.^5^-8) In the early 1990's, when MRI became widely available, its value for the diagnosis of LDH was discussed,^1^,9) but few studies have been undertaken recently. Several papers published in the 1990s concluded that CT-myelograms are more reliable for the diagnosis of LDH than MRI.^1^,^5^,^9^-^11^) Nevertheless, a re-evaluation of the diagnostic value of MRI was required, because of the invasive nature of CT-myelography and because the image qualities of recently produced MRI machines are significantly better than those of machines produced in the 1990s.

It is our clinical experience that the radiologic diagnosis of LDH is not as straightforward as the diagnosis of central or paracentral disc herniations, for the following reasons. First, LDHs, particularly extraforaminal herniations, are easily overlooked (even when their existence is obvious on MR images) because this area may be omitted during image review as neural compression in this area is uncommon. Second, even when extraforaminal areas are carefully observed and a suspicious lesion is detected, the MRI findings may not be confirmative because the lateral regions, particularly in the extraforaminal regions, have more diverse anatomic features than lesions in the central or paracentral regions. Moreover, the extraforaminal regions have no reliable anatomic landmarks on sagittal images,12) which frequently makes even the differentiation of normal and herniated discs challenging. Third, it is often difficult to differentiate LDH from an abnormal nerve root course, nerve root ganglion swelling, neurofibroma, schwannoma, lipoma, or a metastatic tumor. Fourth, a small upper portion of the pedicle seen in axial views could be misinterpreted as a foraminal herniation, particularly when this finding is encountered unilaterally due to an asymmetrical axial cut.12) Fifth, even definitive simple MRI findings on occasion are not correlated with symptoms, e.g., a definitive LDH may be substantially asymptomatic. Finally, it is difficult to discern the real cause of pain in cases of concurrent paracentral disc herniation or spinal stenosis and LDH in the subjacent level.

Park et al.12) concluded that even high quality simple MR images are often inadequate for diagnosing LDH. In the current study, we used inter- and intra-observer MRI agreements as indices of reliability for the diagnosis of LDH, and surprisingly, the resulting kappa values were even lower than we had expected. In fact, the overall inter-observer agreement kappa values averaged only 0.234 for first reviews, and did not improve during second reviews, when they averaged a disappointing 0.166. These figures indicated only slight to fair agreement. On the other hand, intra-observer agreement kappa values averaged 0.479, which still only represented moderate agreement. These findings demonstrate that the reliability of simple MRI is inadequate for the diagnosis of LDH.

The inter- and intra-observer reliability of MRI may appear unexpectedly low, since in our daily practice we can meet patients with an LDH that is clearly observed and easily diagnosed with simple MRI. It is clear that if we analyze the inter- and intra-observer reliability of the MR images of these patients, these values will be higher compared with those in our study. However, they clearly will not be the "true" reliability values of MRI, because the analysis will automatically exclude the patients in whom the diagnosis is not easy. Clearly, true reliability values should be obtained from all the patients including those in whom diagnosis was not confirmed as well as those who are confirmed to have or not to have LDH. Therefore, in our analysis we included all the patients in whom LDH was suspected in a given period of time, whether it was confirmed or not, in order to more fairly analyze the reliability of MRI reflecting the situation as it actually exists. As a result, we found that the reliability of MRI was not satisfactory, as described above.

It should be noted that the low degree of agreement apparent in this study cannot be attributed to poor image quality, an inadequate number of MR images or imaging areas, or to the use of unqualified image reviewers. The MRI machine used was new and installed just before commencement of the study, and between four and six images per level, including all foraminal levels for axial images, were available according to our standard protocol. In addition, the sagittal images included the foraminal zone to the lateral end and the medial portion of the extraforaminal zone. Moreover, all three reviewers specialized in spinal surgery and had several years of experience as assistant professors at university hospitals after completing spinal fellowship training. Thus, our findings indicate that adequate numbers of images and sectioning areas obtained using even modern MRI units do not provide sufficient data to diagnose LDH. Accordingly, we advise that careful re-examination and usage of additional diagnostic modalities, such as coronal imaging, enhanced MRI, MR-discography, enhanced CT, or CT-discography, should be considered when a diagnosis is unclear by simple MRI.

We suggest that additional studies be undertaken to identify MRI findings that suggest LDH, and that other imaging protocols (i.e., coronal images) should be added to enhance the reliability of simple MRI for the diagnosis of LDH. In addition, a comparative analysis of the diagnostic merits of imaging modalities, such as MRI, enhanced MRI, MR-discography, CT, enhanced CT, and CT-discography, should be performed with a view toward devising a practical diagnostic procedure that overcomes the low diagnostic value of simple MRI.

In conclusion, our findings indicate that simple MRI is not a reliable imaging modality for the diagnosis of lumbar LDH, and that some other imaging modality with improved diagnostic values is required.

## Figures and Tables

**Table 1:**
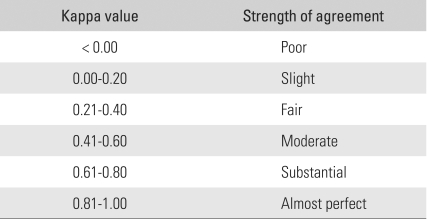
Criteria of Landis and Koch3) for Interpretation of the Strength of Agreement Determined with the Kappa Value

**Table 2:**
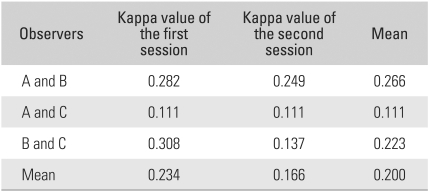
Overall Kappa Values for Inter-observer Reliability

**Table 3:**
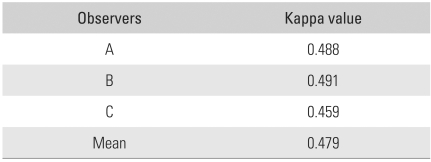
Overall Kappa Values for Intra-observer Reliability

**Table 4:**
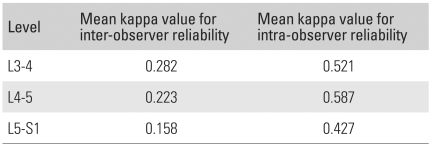
Kappa Values for Inter- and Intra-observer Reliability at Each Level
